# Peripherally administered cisplatin activates a parvocellular neuronal subtype expressing arginine vasopressin and enhanced green fluorescent protein in the paraventricular nucleus of a transgenic rat

**DOI:** 10.1186/s12576-020-00764-z

**Published:** 2020-07-10

**Authors:** Yasuki Akiyama, Mitsuhiro Yoshimura, Hiromichi Ueno, Kenya Sanada, Kentaro Tanaka, Satomi Sonoda, Haruki Nishimura, Kazuaki Nishimura, Yasuhito Motojima, Reiko Saito, Takashi Maruyama, Keiji Hirata, Yasuhito Uezono, Yoichi Ueta

**Affiliations:** 1 Department of Surgery 1, School of Medicine, University of Occupational and Environmental Health, Kitakyushu, 807-8555 Japan; 2 Department of Physiology, School of Medicine, University of Occupational and Environmental Health, 1-1 Iseigaoka, Yahatanishi-ku, Kitakyushu, 807-8555 Japan; 3 Division of Cancer Pathophysiology, National Cancer Center Research Institute, Chuo-ku, Tokyo, 104-0045 Japan

**Keywords:** Arginine vasopressin, Cisplatin, Enhanced green fluorescent protein, Fos B

## Abstract

Cisplatin is one of the most potent anti-cancer drugs, though several side effects can induce stress responses such as activation of the hypothalamic–pituitary adrenal (HPA) axis. Arginine vasopressin (AVP) and corticotrophin-releasing hormone (CRH) expressed in the parvocellular division of the paraventricular nucleus (pPVN) play an important role in the stress-induced activation of the HPA axis. We aimed to evaluate whether intraperitoneal (i.p.) administration of cisplatin could activate parvocellular neurons in the pPVN, using a transgenic rat model that expresses the fusion gene of AVP and enhanced green fluorescent protein (eGFP). Along with the induction of FosB, a marker of neuronal activation, i.p. administration of cisplatin significantly increased eGFP fluorescent intensities in the pPVN. In situ hybridization histochemistry revealed that AVP-eGFP and CRH mRNAs in the pPVN were increased significantly in cisplatin-treated rats. These results suggest that cisplatin administration increases neuronal activation and upregulates AVP and CRH expression in the pPVN.

## Background

Recent advances in anti-cancer drug therapy have improved patients’ survival rates; however, various side effects such as fatigue, nausea, and vomiting occur frequently during chemotherapy. In particular, cisplatin, a widely used anti-cancer drug administered to inhibit the replication of DNA [1–3], can induce stress responses in patients with cancer. We experience clinical cases occurring side effect in the chronic phase after peripheral injection of cisplatin (e.g., delayed nausea/vomiting and anorexia). However, to our knowledge, there is no report to examine the relationship between cisplatin treatment and chronic stress response. In this study, we focused on arginine vasopressin (AVP) in the hypothalamus, which is known to be secreted under chronic stressful condition [4] and related to anorexia [5].

Various kinds of stressful conditions cause activation of the hypothalamic–pituitary adrenal (HPA) axis, inducing an important neuroendocrine response to stress [6]. A primary stress response in neuroendocrine cells that synthesize corticotrophin-releasing hormone (CRH) in the parvocellular division of the paraventricular nucleus (pPVN) of the hypothalamus stimulates adrenocorticotropic hormone (ACTH) release from the anterior pituitary gland via CRH type 1 receptor activation [7]. AVP, as well as CRH, is also known to be synthesized with CRH in the parvocellular neuroendocrine cells of the pPVN; this leads to stimulated release of ACTH in the anterior pituitary gland via activation of the vasopressin receptor 1b (V1b) [8].

In this study, we aimed to determine the cellular and molecular mechanisms through which cisplatin induces changes in AVP expression in the pPVN of rats. First, we focused on characterizing the effects of intraperitoneal (i.p.) administration of cisplatin on AVP synthesis in the pPVN, using a transgenic rat model that expressed the fusion gene of the AVP and the enhanced green fluorescent proteins (eGFP) [9]. This AVP-eGFP transgenic rat is a useful animal model to evaluate the hypothalamic synthesis of AVP; increased AVP expression in reflected by changes in eGFP fluorescent intensity under various stressful conditions [10–13]. A past study showed that eGFP fluorescent intensity in the pPVN was increased dramatically after bilateral adrenalectomy (ADX) and this upregulation could be reversed by dexamethasone administration in AVP-eGFP transgenic rats [11].

Second, we determined whether cisplatin treatment would increase activity of AVP-eGFP-expressing neurons in the transgenic rats through immunohistochemistry, using FosB as a marker of neuronal activity. Previous studies have indicated that FosB is a useful marker for assessing changes in neuronal activity induced by chronic osmotic stimulation and stressful conditions in the pPVN of rats [14, 15]. In this study, we performed bilateral ADX to confirm the same reaction occurs as previously reported [15], using AVP-eGFP transgenic rats.

Finally, we examined the gene expression of AVP, eGFP, and CRH in the pPVN after i.p. administration of cisplatin to determine whether or not it induced the upregulation of AVP-eGFP synthesis in transgenic rats.

Our previous studies demonstrated that i.p. administration of cisplatin caused anorexia-like symptoms for several days in rats [16–18], and gastric effects related to anorexia in rats even though rats have no emetic reflex [19, 20]. Thus, we used this animal model to evaluate whether peripheral administration of cisplatin can affect neuroendocrine cells that synthesize AVP and CRH in the pPVN, using AVP-eGFP transgenic rats.

**Fig. 1:**
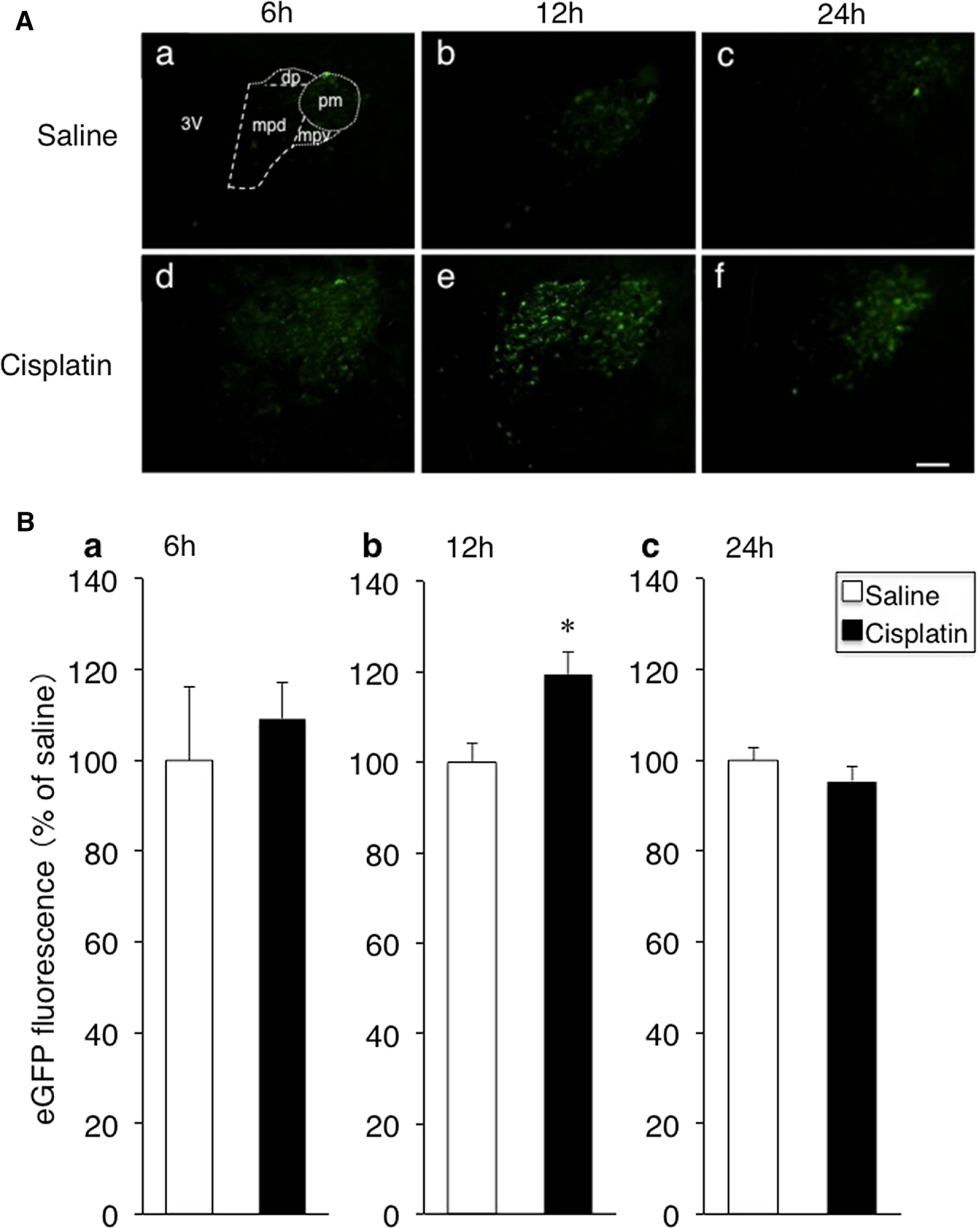
**A** Effects of intraperitoneal (i.p.) administration of cisplatin on arginine vasopressin (AVP)-enhanced green fluorescent protein (eGFP) expression in the paraventricular nucleus (PVN) of AVP-eGFP transgenic rats. Representative eGFP fluorescent microphotographs are shown in the PVN 6, 12 and 24 h after i.p. administration of saline (**A** a–c) or cisplatin (**A** d–f). The PVN consists with at least three parts which contain magnocellular neurosecretory cells in the posterior magnocellular (pm) and medial parvicellular ventral (mpv) parts, pre-autonomic neurons in the dorsal parvicellular (dp) parts, and parvocellular neurosecretory cells in the medial parvicellular dorsal (mpd) parts. The pPVN indicates the mpd part of the PVN containing parvocellular neurosecretory cells. 3V, third ventricle. **B** The eGFP fluorescent intensities in the parvocellular division of the PVN 6, 12, and 24 h after i.p. administration of saline or cisplatin (**B** a–c). Data are presented as the mean ± SEM (for each group at each time point, *n* = 6). *P < 0.05 versus saline-treated group. Scale bar = 100 µm

The PVN consists with at least three parts which contain magnocellular neurosecretory cells in the posterior magnocellular (pm) and medial parvicellular ventral (mpv) parts projecting their axon terminals to the posterior pituitary, pre-autonomic neurons in the dorsal parvicellular (dp) parts, and parvocellular neurosecretory cells in the medial parvicellular dorsal (mpd) parts projecting their axon terminal to the external layers of the median eminence (Fig. 1Aa) [10, 21]. In the present study, the pPVN indicates the mpd part of the PVN containing parvocellular neurosecretory cells.

## Methods

### Animals

Adult male AVP-eGFP Wister transgenic rats [9] (weighing 190–260 g) were used for all experiments. The rats were housed in plastic cages under standard conditions in a colony room maintained at 23–25 °C, with a 12:12 h light:dark cycle (lights on at 7:00 a.m.). The animals were fed a standard rat diet and tap water ad libitum. All procedures were performed in accordance with the ethical standards of the institution where the studies were conducted and had full ethical approval by the Ethics Committee of Animal Care and Experimentation, University of Occupational and Environmental Health, Japan (AE00-007).

### Reagents

Cisplatin (*cis*-diammineplatinum (II) dichloride) (P-4394, Sigma-Aldrich Japan Co. LLC., Tokyo, Japan) was dissolved in 0.9% sterile physiological saline (Otsuka Pharmaceutical Co. LTD., Tokyo, Japan) in a warm water bath (60 °C).

### Surgical procedures

According to our previous study [11], dorsal bilateral ADX and sham operations were performed on AVP-eGFP Wistar transgenic rats under deep anesthesia (sodium pentobarbital 50 mg/kg body weight, i.p. injection). The animals (bilateral ADX rats (*n* = 6) and sham-operated rats (*n* = 2)) were returned to their cages. After surgery, both groups were given a solution of 0.9% NaCl to drink ad libitum.

## Experimental procedures

### Quantification of AVP-eGFP fluorescent intensity and immunohistochemical FosB expression

AVP-eGFP transgenic rats were administered cisplatin (6 mg/kg, i.p.) (*n* = 22) or vehicle (*n* = 20). They were divided into seven groups: control (0 h; baseline), 6, 12, and 24 h after i.p. administration of cisplatin or saline (*n* = 6–8 in each group). Our previous study showed that i.p. administration of cisplatin induced anorexia and activation of oxytocin pathway and nesfatin-1 signals [16–18]. In these previous studies, a dose of injected cisplatin was 6 mg/kg. Thus, we used same dose of cisplatin (6 mg/kg) in this study.

The bilateral ADX and sham-operated rats were deeply anesthetized by an i.p. administration of sodium pentobarbital (50 mg/kg), and perfused transcardially with 100 mL of 0.1 M phosphate buffer (PB, pH 7.4) containing heparin (1000 U/L), and 150 mL of fixative solution containing 4% paraformaldehyde (PFA) in 0.1 M PB.

At 24 h after operation, the brains were removed immediately after perfusion, and then divided into small blocks, including the hypothalamus. The block was post-fixed with 4% PFA in 0.1 M PB for 48 h at 4 °C. Tissues were then cryoprotected in 20% sucrose in 0.1 M PB for 24 h. Serial sections (30 μm tick) were cut using a microtome (REM700; Yamato Kohki Industrial Co., Ltd., Saitama, Japan). Sections then washed twice with 0.1 M phosphate-buffered saline (PBS), followed by in 0.1 M Tris buffer (pH 7.6) containing 0.3% Triton X-100. Floating sections were incubated with 1% hydrogen peroxide for 60 min, followed by a rabbit monoclonal anti-FosB protein antiserum (ab11919, Abcam) diluted at 1:1500 in 0.1 M PBS containing 0.3% Triton X-100 at 4 °C for 4 days. After being washed in 0.3% Triton X-100/PBS for 20 min, the floating sections were further incubated for 120 min with a biotinylated secondary antibody solution (1:250), followed by amplification with an avidin–biotin–peroxidase complex (Vector Laboratories Inc., Burlingame, CA, USA) for 120 min. At first, the slices including the PVN were selected by referring the rat brain atlas [22]. After that, we observed the PVN with microscope and the mPVN and pPVN were distinguished by morphologically, such as their containing cell size and their location. After determining those analyzing area, we observed eGFP fluorescence in the PVN and captured the images by fluorescence microscopy (ECLIPSE Ti-E; Nikon, Tokyo, Japan) with a GFP filter (Nikon) for assessing the AVP-eGFP fluorescence and RFP filter for assessing the FosB expression. Fluorescent microscopic images for two sections of the PVN were obtained from each rat using a digital camera (DS-L2, DS-Fi1; Nikon Corp.). Then, eGFP fluorescent intensities in the PVN were measured by the imaging analysis system (NIS-Elements; Nikon), as previously described [10, 12]. The number of FosB-immunoreactive (ir) cells in the PVN was counted manually for each image.

### In situ hybridization for AVP, eGFP, and CRH mRNAs

To analyze changes in AVP, eGFP, and CRH mRNA levels in the pPVN, AVP-eGFP transgenic rats were divided into five groups depending on the timing of drug administration: control (0 h; baseline), 6 h, or 12 h after i.p. administration of cisplatin (6 mg/kg) or saline (*n* = 12 in each group). Animals were all decapitated at the same time of day (13:00–14:00); the brains were removed quickly after decapitation and put on the dry ice.

The frozen sections (12 μm) were hybridized with ^35^S-labeled oligonucleotide probes with sequences complementary to the AVP, eGFP, and CRH genes. These probes used for in situ hybridization have been characterized and described in our previous studies [10, 12, 13]. After in situ hybridization, the signals in the pPVN were analyzed using Image J software (National Institutes of Health (NIH), USA). In particular, AVP mRNA signals obtained from the pPVN were analysis carefully according to previous studies [23, 24].

### Measurement of plasma concentrations of corticosterone

Truncal blood samples were collected from decapitated rats 6 and 12 h after i.p. administration of saline or cisplatin (*n* = 12 in each group). Plasma concentrations of corticosterone were analyzed by enzyme-linked immune sorbent assay (ELISA) (Corticosterone ELISA Kit, Cayman chem., MI, USA).

### Statistical analysis

All data are presented as the mean ± standard error of mean (S.E.M.). Student *t* tests were used for statistical comparisons between groups. *P *< 0.05 was considered statistically significant.

## Results

### Quantification of eGFP intensity in the pPVN after i.p. administration of cisplatin

The representative images captured on a fluorescent microscopic show marked increases in eGFP fluorescence in the pPVN 12 h after i.p. administration of cisplatin compared with pre-injected baseline (0 h) (Fig. 1Ae). Compared with saline treatment, i.p. administration of cisplatin significantly increased the eGFP fluorescent intensities in the pPVN 12 h (Fig. 1A, Bb), but not 6 h or 24 h (Fig. 1A, Ba, c) after injection.

### FosB expression in the pPVN after bilateral ADX and i.p. administration of cisplatin

**Fig. 2:**
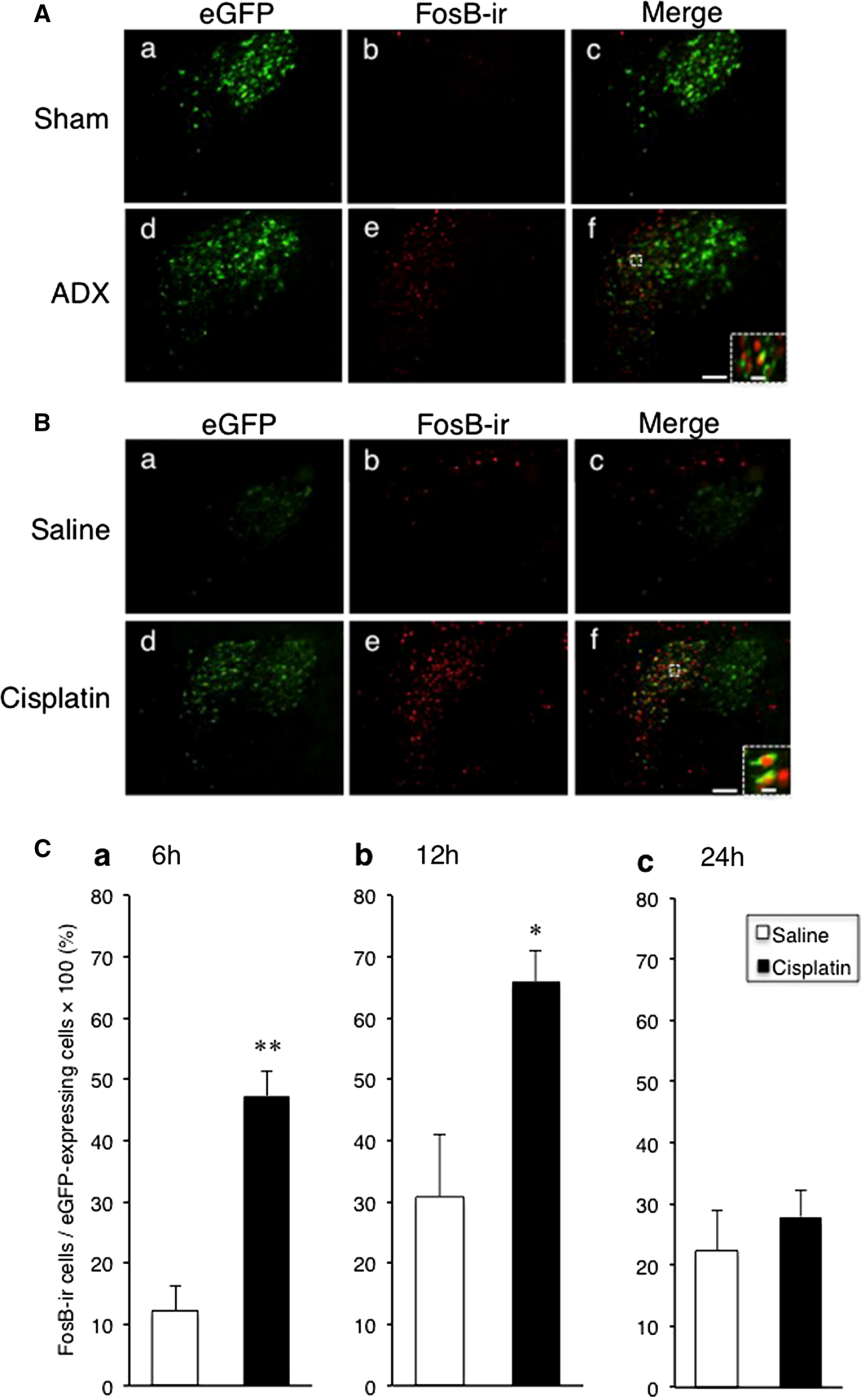
**A** Arginine vasopressin (AVP)-enhanced green fluorescent protein (eGFP) and FosB-immunoreactivity (ir) in the paraventricular nucleus (PVN) 24 h after sham operation (**A** a–c) or bilateral adrenalectomy (ADX) (A d, e, f) in AVP-eGFP transgenic rats. Fluorescent microphotographs of the eGFP fluorescence and FosB-ir in the parvocellular division of the PVN are merged in **A** c, f. Scale bar = 100 µm. The scale bar of the inset in **A** f = 5 µm. **B** The eGFP fluorescence and FosB-ir in the PVN 12 h after i.p. administration of saline (**B** a–c) or cisplatin (**B** d–f) in AVP-eGFP transgenic rats. Fluorescent microphotographs of the eGFP fluorescence and FosB-ir in the parvocellular division of the PVN are merged in **B** c, f. Scale bar = 100 µm. The scale bar of the inset in **A** f = 5 µm. **C** The percentage of eGFP-expressing cells co-expressing FosB-ir in the parvocellular division of the PVN of AVP-eGFP transgenic rats 6, 12, and 24 h after i.p. administration of saline (**B** a–c) or cisplatin (**B** d–f) in AVP-eGFP transgenic rats. Data are presented as the mean ± SEM (for each group at each time point, *n* = 6). *P < 0.05, **P < 0.01 versus saline-treated group

The eGFP fluorescent intensities in the pPVN were markedly increased 24 h after bilateral ADX compared with sham operation (Fig. 2Aa, d). Fluorescent immunohistochemistry revealed that FosB-ir were observed in almost all eGFP-expressing cells in the pPVN (Fig. 2Ae, f) of transgenic rats following bilateral ADX, consistent with the results of a previous study [15]. The number of eGFP-expressing cells exhibiting FosB-ir in the pPVN increased significantly 6 h and 12 h, but not 24 h, after i.p. administration of cisplatin compared with saline-treated controls (Fig. 2B, Ca–c).

### AVP mRNA, eGFP mRNA, and CRH mRNA in the pPVN after i.p. administration of cisplatin

**Fig. 3:**
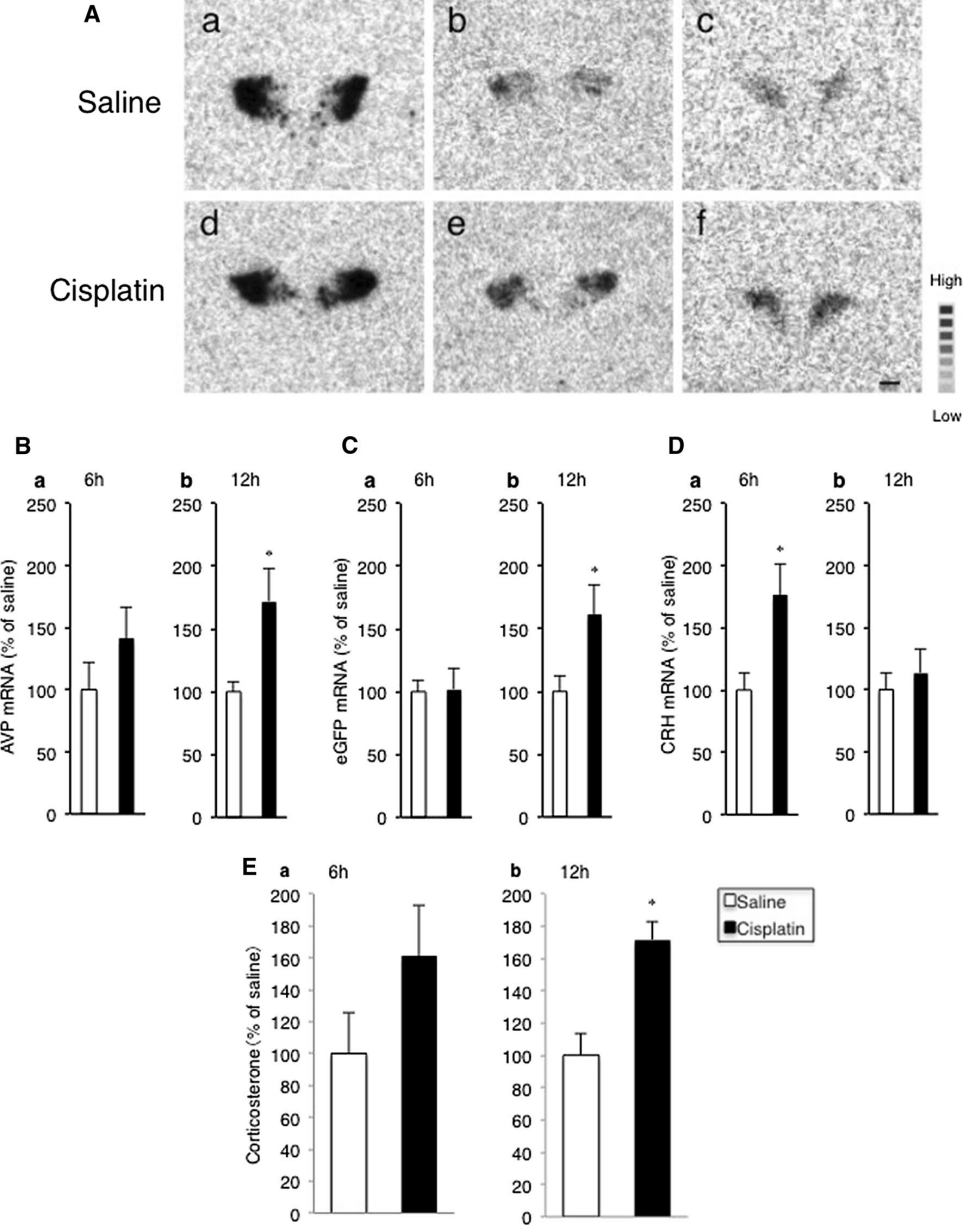
**A** Representative autoradiographic images of the hybridized with ^35^S-labeled oligodeoxynucleotide probes complimentary to AVP mRNA (**A** a, d), eGFP mRNA (**A** b, e), and CRH mRNA (**A** e, f) in the paraventricular nucleus (PVN) of AVP-eGFP transgenic rats. Sections were obtained 12 h after i.p. administration of saline (**A** a, b) or cisplatin (**A** d, e), and 6 h after i.p. administration of saline (**A** c) or cisplatin (**A** f). The highest signal intensity is indicated in black and the lowest signal intensity is indicated in white. Scale bar = 100 µm. AVP mRNA (**B**), eGFP mRNA (**C**), and CRH mRNA (**D**) were measured in the parvocellular division of the PVN 6 and 12 h after i.p. administration of saline or cisplatin in AVP-eGFP transgenic rats. Data are presented as the mean ± SEM (for each group at each time point, *n* = 12). *P < 0.05 versus saline-treated group. **E** Plasma concentrations of corticosterone 6 and 12 h after i.p. administration of saline or cisplatin in AVP-eGFP transgenic rats. Data are presented as the mean ± SEM (for each group at each time point, *n* = 12). *P < 0.05 versus saline-treated group

The levels of AVP mRNA and eGFP mRNA in the pPVN increased significantly 12 h, but not 6 h, after i.p. administration of cisplatin compared with saline-treated controls (Fig. 3Aa, b, d, e, Ba, b, Ca, b). CRH mRNA levels in the pPVN increased significantly 6 h, but not 12 h, after i.p. administration of cisplatin compared with saline treatment (Fig. 3Ac, f, Da, b).

### Plasma concentrations of corticosterone

Plasma concentrations of corticosterone were increased significantly 12 h but not 6 h after i.p. administration of cisplatin compared with saline-treated controls (Fig. 3Ea, b).

## Discussion

The present study provided the first evidence that parvocellular neurons in the pPVN upregulate the synthesis of AVP and CRH with the induction of FosB following i.p. administration of cisplatin.

The pPVN contains neuroendocrine cells that synthesize CRH and AVP, as well as pre-autonomic neurons that project their axon terminals to the preganglionic autonomic neurons in the brainstem and the spinal cord [25]. Under stressful condition, the pPVN synthesizes and secretes CRH and AVP. They are released into the hypophysial portal vessels that access on the anterior pituitary gland. Binding of CRH and AVP to its receptors on pituitary corticotropes induces the release of ACTH into the systemic circulation [6].

AVP and CRH in the pPVN play an important role in the stress-induced activation of the HPA axis. CRH synthesis in the pPVN is increased acutely in response to a stressor, while AVP synthesis in the pPVN is upregulated later under chronic stress conditions, suggesting that AVP and CRH may differentially [4] modulate stress responses. Peripheral administration of cisplatin may not stimulate parvocellular neurons in the pPVN directly, as indicated, secondary effects, including side effects such as emetic reactions, may activate parvocellular neurons in the pPVN. Cisplatin induces not only nausea/vomiting but also other side effects such as syndrome of inappropriate antidiuretic hormone (SIADH) and impaired cognitive functions [26–31]. The possible mechanisms of cisplatin-induced SIADH are as follows: (1) cisplatin-induced vomiting/hypovolemia may stimulate AVP synthesis/secretion, (2) cisplatin may activate AVP neurons in the hypothalamus directly, and (3) continuous secretion of AVP into the portal vein in the external layer of the median eminence projected from the pPVN [26, 27]. Cisplatin-induced impairment of learning and memory may be caused by direct toxic effects on neurons, in particular hippocampal neurons [28–30]. It is unclear whether CRH pathway may be involved in their effects [31]. These possibilities should be determined by further study. In the present study, CRH mRNA levels increased significantly 6 h after i.p. administration of cisplatin, while AVP mRNA was increased 12 h after administration, consistent with reports that CRH is related to the acute phase, while AVP is related to the chronic phase of stress responses [32, 33]. Another possibility is that cisplatin may suppress adrenal cortical function to secrete glucocorticoids directly. Our previous study [10] demonstrated that bilateral ADX induced a robust increase in eGFP fluorescence in CRH-ir neurons in the pPVN using the same AVP-eGFP transgenic rat model. A previous study showed that the increased expression of AVP-eGFP in the pPVN after bilateral ADX was suppressed significantly by treatment with dexamethasone, a corticosteroid [11]. In addition, our preliminary study showed that plasma corticosterone levels were not increased significantly 30 and 90 min after i.p. administration of cisplatin. Although chemotherapy could be stressful in cancer patients, the reason why plasma cortisol levels in cancer patients decreased after chemotherapy [34] is unclear and requires further investigation. However, we were able to show a significant increase in plasma corticosterone levels 12 h after i.p. administration of cisplatin administration. There is one explanation that late increased AVP synthesis in the pPVN may be involved in the chronic phase of stress response.

In the present study, we used immunohistochemistry for FosB to infer changes in the amount of neuronal activity in the pPVN after i.p. administration of cisplatin. Although FosB is one of the immediate early genes, Fos induction is typically used as a marker of neuronal activity after exposure to various stressful conditions [35]. Our preliminary study showed that Fos-ir is not detectable in the pPVN 6 h and 12 h after i.p. administration of cisplatin, which is understandable, as the peak of Fos induction occurs between 90 min and 120 min. Thus, we instead used FosB, a known marker that is useful for detecting changes in neuronal activity under chronic stress conditions [14, 15].

The pathophysiological explanation for the induction of FosB and the increase in AVP synthesis in the pPVN after i.p. administration of cisplatin remains obscure. However, there are several hypotheses that could explain these observations. For example, various parts of the brain, including the PVN, are involved in regulating feeding behavior [36]. Our previous studies demonstrated that oxytocin pathway and nesfatin-1 signals in the central neurons system were related to anorexia-like behavior after i.p. administration of cisplatin in rats [17, 18]. Several studies also showed that AVP and CRH-expressing neurons in the hypothalamus were involved in the regulation of appetite behavior [5, 37, 38]. Peripherally administered cisplatin may activate inhibitory neuronal circuits involved in these feeding behaviors, including AVP and other neuropeptides in the central nervous system. On the other hand, cisplatin may also act on the chemoreceptor trigger zone in the brainstem, the afferent vagal nerves, and at sites within the digestive tract that is related to emetic responses that can be induced by chemotherapy. Although anti-emetic drugs are used clinically, it has still been difficult to prevent emesis completely in patients undergoing cancer chemotherapy [39–41].

## Conclusions

In conclusion, peripheral administration of cisplatin induced an upregulation of AVP synthesis and increased neuronal activity in the pPVN, which may lead to enhanced activation of the HPA axis. Our findings may provide insights for developing novel therapeutic strategies to combat the side effects of anti-cancer drugs.
